# Novel C_60_ Fullerenol-Gentamicin Conjugate–Physicochemical Characterization and Evaluation of Antibacterial and Cytotoxic Properties

**DOI:** 10.3390/molecules27144366

**Published:** 2022-07-07

**Authors:** Aleksandra Nurzynska, Piotr Piotrowski, Katarzyna Klimek, Julia Król, Andrzej Kaim, Grazyna Ginalska

**Affiliations:** 1 Chair and Department of Biochemistry and Biotechnology, Medical University of Lublin, Chodzki 1 Street, 20-093 Lublin, Poland; aleksandra.nurzynska@umlub.pl (A.N.); g.ginalska@umlub.pl (G.G.); 2 Department of Chemistry, University of Warsaw, Pasteura 1 Street, 02-093 Warsaw, Poland; j.krol10@student.uw.edu.pl (J.K.); akaim@chem.uw.edu.pl (A.K.)

**Keywords:** functionalized fullerenes, fullerenols, antibiotics, gentamicin, antibacterial properties, cytotoxicity, skin fibroblasts, nanomedicine

## Abstract

This study aimed to develop, characterize, and evaluate antibacterial and cytotoxic properties of novel fullerene derivative composed of C_60_ fullerenol and standard aminoglycoside antibiotic–gentamicin (C_60_ fullerenol-gentamicin conjugate). The successful introduction of gentamicin to fullerenol was confirmed by X-ray photoelectron spectroscopy which together with thermogravimetric and spectroscopic analysis revealing the formula of the composition as C_60_(OH)_12_(GLYMO)_11_(Gentamicin)_0.8_. The dynamic light scattering (DLS) revealed that conjugate possessed ability to form agglomerates in water (size around 115 nm), while Zeta potential measurements demonstrated that such agglomerates possessed neutral character. In vitro biological assays indicated that obtained C_60_ fullerenol-gentamicin conjugate possessed the same antibacterial activity as standard gentamicin against *Staphylococcus aureus*, *Staphylococcus epidermidis*, *Pseudomonas aeruginosa*, and *Escherichia coli*, which proves that combination of fullerenol with gentamicin does not cause the loss of antibacterial activity of antibiotic. Moreover, cytotoxicity assessment demonstrated that obtained fullerenol-gentamicin derivative did not decrease viability of normal human fibroblasts (model eukaryotic cells) compared to control fibroblasts. Thus, taking into account all of the results, it can be stated that this research presents effective method to fabricate C_60_ fullerenol-gentamicin conjugate and proves that such derivative possesses desired antibacterial properties without unfavorable cytotoxic effects towards eukaryotic cells in vitro. These promising preliminary results indicate that obtained C_60_ fullerenol-gentamicin conjugate could have biomedical potential. It may be presumed that obtained fullerenol may be used as an effective carrier for antibiotic, and developed fullerenol-gentamicin conjugate may be apply locally (i.e., at the wound site). Moreover, in future we will evaluate possibility of its applications in *inter alia* tissue engineering, namely as a component of wound dressings and implantable biomaterials.

## 1. Introduction

The fullerenes, a family of carbon allotropes, represent very promising group of chemical molecules in the context of biomedical applications [1,2,3,4,5]. In this case, their hydrophilic derivatives have been gained the special scientific attention, thanks to water solubility [2] and biological activities, such as anti-oxidative and radical-quenching [6,7], antibacterial [8,9], antiviral [10,11], DNA photocleavage [12,13] as well as enzyme-inhibiting [14,15,16] or neuroprotective [17,18] properties. Thus, thanks to aforementioned features, it was suggested that water-soluble fullerene derivatives could be potentially used as antioxidant agents, therapeutics for the treatment of HIV infection, and also as photosensitizers or drug carriers [1,3,19,20].

The applications of water-soluble fullerenes as drug delivery vehicles are especially focused on anticancer therapy [1,3,21,22]. For instance, Chaudhuri et al. [23] demonstrated that a very popular chemotherapeutic agent–doxorubicin (DOX) together with polyethylene glycol (PEG) may be conjugated with polyhydroxylated fullerene (PHF) C_60_, which allows to obtain nanocomplex with high therapeutics efficacy and lower cytotoxicity compared to free doxorubicin. PHFs were also linked with other anti-cancer drug, namely methotrexate (MTX). Bahuguna et al. [24] received FLU-MTX nanocomplex, which was characterized by increased availability of drug to the biological system and increased cytotoxicity toward cancer cells compared to free MTX. In turn, Prylutska et al. [25] developed anti-cancer nanocomplex consisted of C_60_ fullerene aqueous colloid solution (C_60_FAS) and cisplatin (Cis). The authors showed that C_60_ + Cis nanocomplex exhibited higher toxicity towards human leukemia cells compared to cisplatin alone. Moreover, in vivo study with Lewis lung carcinoma (LLC) C57BL/6J male mice demonstrated that C_60_ + Cis conjugate inhibited tumor growth more potently than free cisplatin.

Nevertheless, to the best of our knowledge, there are no research reports which present adducts composed of fullerenols and antimicrobials. Such composition seems to be promising thanks to biological activities of fullerenols and antimicrobial agents. In other words, we assumed that C_60_ fullerenol will be not only a nanocarrier for gentamicin, but also may exhibit beneficial biological properties (for instance antioxidant or free-radical scavenging). It is especially important as an application of antibiotics most often is associated with unfavorable side effects. It is known that aminoglycoside antibiotics (such as gentamicin) may cause nephrotoxicity (damage of kidneys) and ototoxicity (damage of hearing organ) [26,27]. Thus, our preliminary study aimed to develop novel fullerene derivative composed of C_60_ fullerenol and standard aminoglycoside antibiotic–gentamicin. Desired product was synthesized by binding (3-glycidyloxypropyl)trimethoxysilane linker to fullerenol surface using part of its hydroxyl groups and further reaction of introduced oxirane moieties with gentamicin –NH_2_ groups. Synthesized nanomaterial was evaluated using X-ray photoelectron spectroscopy (XPS), Fourier-transform infrared spectroscopy (FT-IR), thermogravimetry (TGA), and dynamic light scattering (DLS). This nanoconjugate was also subjected to initial evaluation of biological properties. The antibacterial activity of fullerenol-gentamicin nanocarrier was assessed towards Gram-positive and Gram-negative bacterial strains (i.e., *Staphylococcus aureus*, *Staphylococcus epidermidis*, *Pseudomonas aeruginosa*, and *Escherichia coli*, respectively). Moreover, its cytotoxicity was estimated using model eukaryotic cells–normal human skin fibroblasts (BJ cell line). To our best knowledge, it is first research, which demonstrates effective method allowing to conjugate fullerenol with aminoglycoside antibiotic.

## 2. Results and Discussion

### 2.1. Characterization of C_60_ Fullerenol-Gentamicin Derivative

Composition of synthesized C_60_ fullerenol-gentamicin conjugate was investigated using X-ray photoelectron spectroscopy (XPS). Survey XPS spectrum obtained for the newly synthesized fullerene derivative revealed the presence of carbon, oxygen, nitrogen, and silicon atoms, namely elements expected for gentamicin functionalized fullerenol (see Supplementary Material, Figure S1).

**Figure 1:**
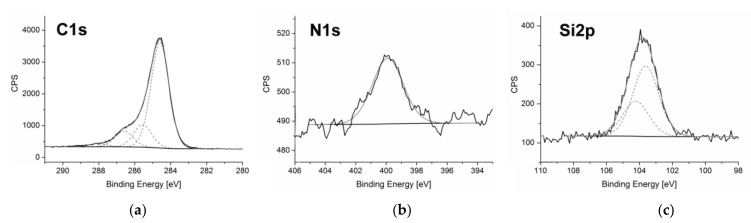
The XPS spectra obtained for C_60_ fullerenol-gentamicin derivative (**60FGG**)–C1s spectrum (**a**), N1s core level region (**b**), and Si2p region (**c**).

C1s spectrum obtained for C_60_ fullerenol-gentamicin derivative (**60FGG**) (Figure 1a) was deconvoluted with very good correlation into four signals [28]. The lowest binding energy peak, centered at 284.6 eV is assigned to sp2 carbon from fullerene cage. Second signal was registered at 285.6 eV and can be attributed to the sp3 carbon. Next contribution (at 286.6eV) was ascribed to C-O carbon atoms from fullerenol, linker, and gentamicin moieties. The highest binding energy peak, arising from C = O carbon atoms, was registered at 288.0 eV and suggests that traces of hydroxyl groups present at the C_60_ surface were oxidized during the synthesis process.

Single peak observed in the N1s core level region (Figure 1b) with maximum located at 399.9 eV is assigned to the nitrogen atoms from amino groups of gentamicin bound to the fullerenol derivative surface. This result confirms successful introduction of aforementioned aminoglycoside antibiotic.

Another strong evidence of the modification of fullerenol surface was provided by Si2p region of XPS spectrum (Figure 1c), where double peak is observed with signals centered at 103.6 and 104.2 eV assigned to 2p3/2 and 2p1/2 silicon atoms from the GLYMO linker [29]. Those signals show expected area ratio of 2:1 and splitting of 1.2 eV.

Additionally, lack of signals associated with sulfur atoms implies that introduced gentamicin molecules were neutralized during the final step of synthesis. Released sulfate ions were removed during the dialysis process.

**Figure 2:**
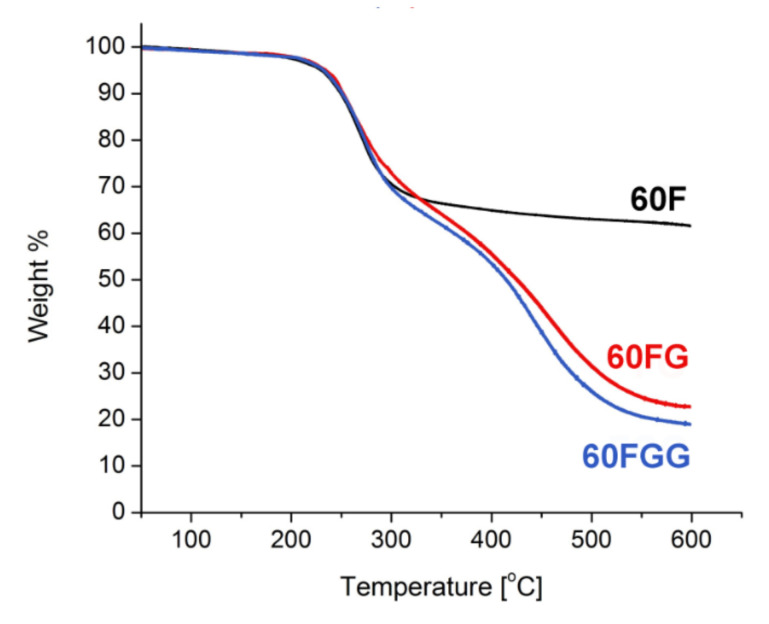
Thermogravimetric analysis of fullerenol (**60F**), GLYMO modified fullerenol (**60FG**), and gentamicin functionalized C_60_ fullerene (**60FGG**) at 5 K/min under N_2_ atmosphere.

The XPS results along with thermogravimetric measurements [30] (Figure 2) allowed to calculate the approximate composition of fullerenol, its GLYMO derivative, and synthesized fullerenol-gentamicin conjugate. Single step thermal decomposition of **60F** resulted in weight loss of 35%, which corresponds to 23 hydroxyl groups. GLYMO functionalized fullerenol and its gentamicin adduct revealed more complex TGA curves, both with shape close to two step decomposition. Total weight loss was determined to be 77% in case of **60FG** and 80% for **60FGG**. Thus, final composition of synthesized nanomaterial was estimated to be C_60_(OH)_12_(GLYMO)_11_(Gentamicin)_0.8._

**Figure 3:**
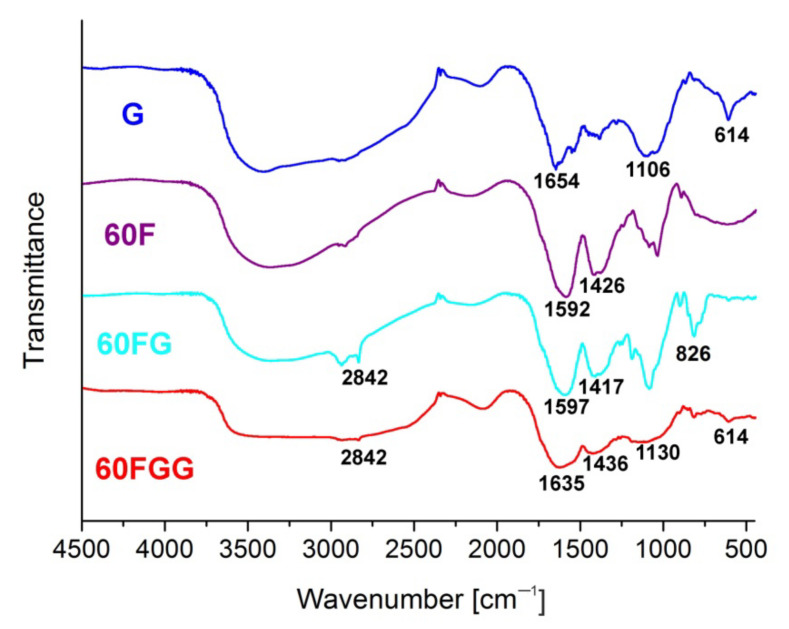
FT-IR spectra recorded in KBr disk for: gentamicin (**G**), fullerenol (**60F**), GLYMO modified fullerenol (**60FG**), and gentamicin functionalized C_60_ fullerene (**60FGG**).

Functionalization of C_60_ fullerenol (**60F**) with GLYMO allowed to form **60FG** and then to introduce gentamicin molecules in order to obtain desired conjugate, i.e., **60FGG** that was also confirmed by means of FT-IR spectroscopy (Figure 3). First of all, **60FGG** spectrum shows broad band from 3700 to 2400 cm^−1^, characteristic to gentamicin substrate. Despite relatively strong intensity of this signal, small peak at 2842 cm^−1^, also observed in **60FG** spectrum is still present, indicating that final product has **60FG** contributions associated with its alkane C-H stretching modes. Additionally, amine N-H bending mode is present at around 1635 cm^−1^ in both gentamicin functionalized fullerene and unmodified gentamicin samples. This band overlaps with signals attributed to C=C stretching vibrations, which are present in obtained fullerene derivatives at around 1600 cm^−1^. Presence of signal centered around 1420 cm^−1^ in all fullerene related samples: **60F**, **60FG** and **60FGG** confirms presence of C_60_ core in those products [31]. Broad band at around 1130 cm^−1^ present in both **60FGG** and **G** samples can be associated with C-O stretching vibrations coming from secondary and tertiary alcohol groups present in the gentamicin structure. Spectrum of **60FG** shows band at 826 cm^−1^, which can be assigned to asymmetric ring deformation of epoxide groups [32,33,34], coming from introduced GLYMO linkers. Intensity of this signal is significantly lowered in the sample after reaction with gentamicin, which is in good agreement with expected reaction pathway, leading to epoxide ring opening due to reaction with amino groups from antibiotic molecules. Another very important signal, which is associated with gentamicin moiety can be observed in **60FGG** spectrum at 614 cm^−1^, additionally confirming presence of the aminoglycoside antibiotic in final product.

**Figure 4:**
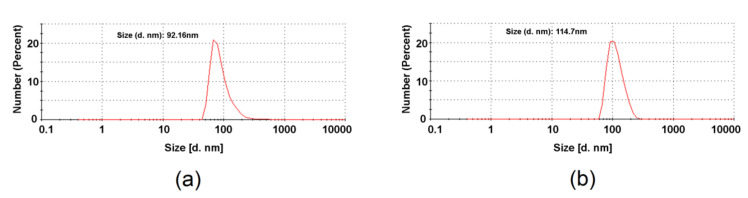
Size distributions obtained from DLS measurements for: fullerenol **60F** (**a**) and C_60_ fullerene gentamicin conjugate **60FGG** (**b**).

The dynamic light scattering (DLS) measurements allowed to determine the hydrodynamic diameter of the synthesized fullerene derivatives in water (Figure 4). Size distribution for **60F** revealed single peak at approximately 92 nm, which is value typical for fullerenol associations [35,36,37]. Introduction of gentamicin onto fullerenol surface results in slight increase of the size of agglomerates, which was found to be around 115 nm.

DLS analysis was accompanied by Zeta potential measurements. Both fullerenol (**60F**) and gentamicin functionalized C_60_ fullerene (**60FGG**) revealed to form neutral agglomerates [38]. Corresponding Zeta potential values were determined to be −0.1 mV and 0.1 V for **60F** and **60FGG**, respectively (see Supplementary Material, Figures S2 and S3). Results obtained for C_60_ fullerenol-gentamicin conjugate are in good agreement with XPS data, which suggested that gentamicin was neutralized during **60FGG** synthesis and purification process.

### 2.2. Antibacterial Properties of Gentamicin Functionalized C_60_ Fullerene Derivative

Firstly, antibacterial tests showed that hydroxylated C_60_ fullerene (**60F**) did not exhibit antibacterial activity towards all tested bacterial strain. Interestingly, some authors indicated that water-soluble C_60_ fullerene derivatives possessed antibacterial activities [8,9]. For instance, Deryabin et al. [8] developed water-soluble C_60_ fullerene derivatives bearing amine (AF) and carboxylic (CF) groups. The authors indicated that both derivatives had ability to form nanoclusters in aqueous solutions–diameters ranged from 2 to 200 nm (AF) and from 70 to 100 nm (CF) (based on DLS measurements). Nevertheless, only AF derivatives possessed antibacterial properties. The authors suggested that this phenomenon was associated with electrostatic interactions between fullerene derivatives and cells. Fullerene derivative (AF) with positive charge adhered to negatively charged bacterial cells, while negatively charged CF derivatives did not possess ability to interact with bacterial cells. Thus, it seems that charge of fullerene derivative has greater influence on antibacterial activity than particle size. In our study, we showed (DLS measurements, Section 2.1) that size distribution of **60F** in water was 92 nm, while introduction of gentamicin onto fullerenol surface resulted in slight increase of the size of agglomerates, which was found to be around 115 nm. Thus, the particle size of both products (**60F** and **60FGG**) was comparable. In turn, Zeta potential measurements showed that both possessed neutral charges. Thus, these results may explain the lack of bacterial properties of **60F**–neutrally charged derivative rather should not have ability to adhere to negatively charged bacterial cells. Our study also demonstrated that both gentamicin sulfate salt (**G**) and conjugate composed of C_60_ fullerenol and gentamicin (**60FGG**) possessed the same activity–values of MIC and MBC for both compounds were identical and ranged from 0.125 to 1 μg/mL, depending on bacterial strain (Table 1 and Table 2). Thus, these results indicated, that conjugate composed of **G** and **60F** did not exhibit greater antibacterial activity than **G** alone, but allowed to maintain the antibacterial properties of **G**. In other words, these antibacterial assays confirmed that newly developed method for fabrication of **60FGG** allows to obtain effective, antibacterial agent. It is worth noting that the aim of this study is not to develop C_60_ fullerenol with antibacterial properties or C_60_ fullerenol-gentamicin conjugate with better activity than gentamicin alone. We aimed to develop nanoconjugate which will maintain antibacterial activity of gentamicin and will not exhibit cytotoxic properties towards eukaryotic cells.

### 2.3. Cytotoxic Properties of Gentamicin Functionalized C_60_ Fullerene

**Figure 5:**
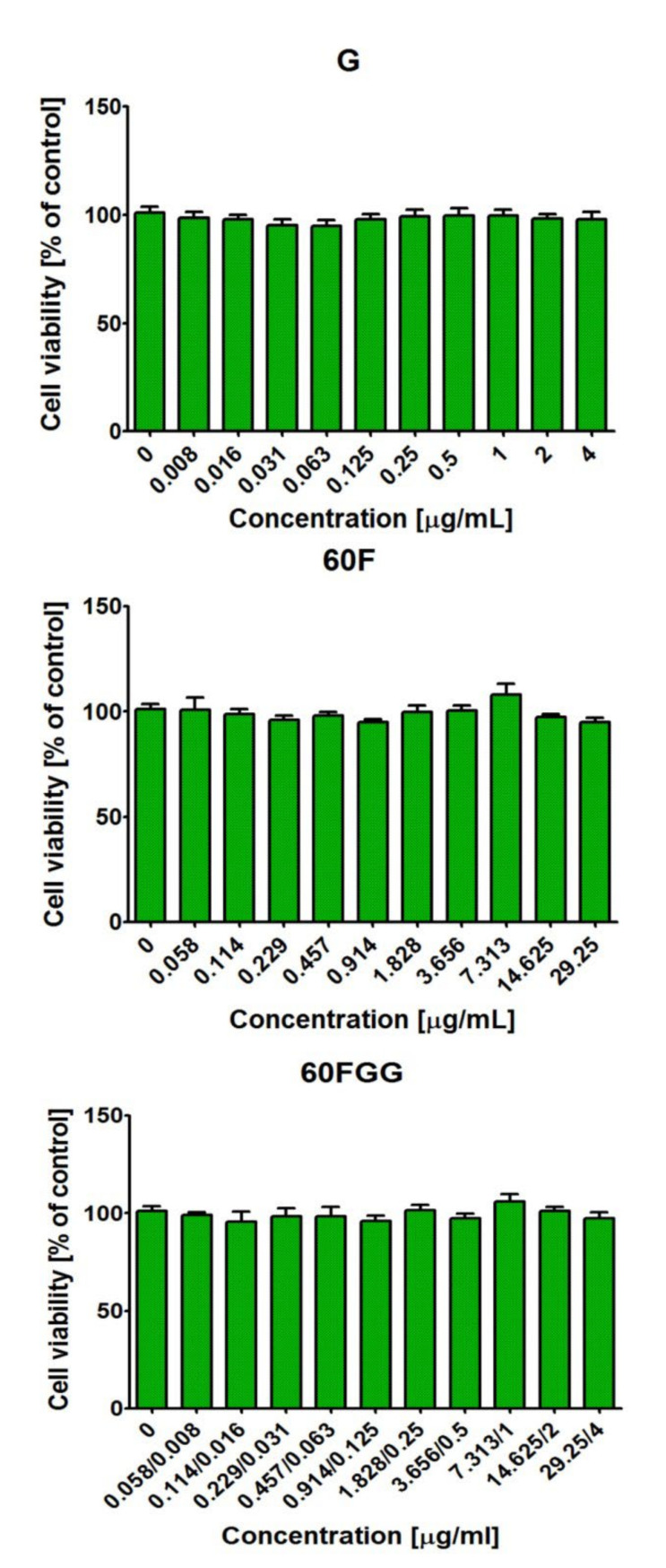
Viability of normal human skin fibroblasts (BJ cell line, CRL-2522^TM^) after 24 h treatment with aqueous solutions of gentamicin sulfate salt (**G**), C_60_ fullerenol (**60F**), and gentamicin functionalized C_60_ fullerene (**60FGG**). Gentamicin (**G**) was evaluated at concentration of 4–0.008 μg/mL, C_60_ fullerenol (**60F**) at concentration of 29.25–0.058 μg/mL, while concentrations of gentamicin and C_60_ fullerenol in obtained conjugate (**60FGG**) ranged from 4–0.008 μg/mL and 29.25–0.058 μg/mL, respectively. The cell viability was assessed by MTT assay. The results were not statistically significant (*p* > 0.05) compared to control, namely cell incubated with culture medium without tested compounds; one-Way ANOVA test, followed by a Tukey’s multiple comparison test.

The MTT assay indicated that all tested solutions prepared from gentamicin sulfate salt (**G**), C_60_ fullerenol (**60F**), and gentamicin functionalized C_60_ fullerene (**60FGG**) were non-cytotoxic towards normal human skin fibroblasts (Figure 5). The cell viability after treatment with tested solutions of **G**, **60F**, and **60FGG** was comparable with viability of control cells (cultured without investigated compounds) and close to 100%. These results indicated that introduction of gentamicin to C_60_ fullerenol did not have unfavorable influence on cell behavior. Thus, obtained **60FGG** conjugate possessed desired antibacterial activity (please see Section 2.2) and at the same time was safe for model eukaryotic cells (human fibroblasts) in vitro.

In general, water-soluble fullerene derivatives exhibit no or minimal cytotoxicity [19]. Nevertheless, it is also known that cytotoxicity of these derivatives may be controlled by *inter alia* derivatization of their surfaces. Thus, the increase in cytotoxicity of fullerene derivatives is suggested as a promising approach in antibacterial or cancer therapies [39]. Aforementioned positive-charged amine derivatives of fullerene (AF) (please see Section 2.2) possessed antibacterial activity thanks to their ability to adhere to negatively charged bacterial cells. Nevertheless, cell membrane of eukaryotic cells are also negative-charged, which suggests that such derivative will also adhere to these cells. Indeed, the authors of cited manuscript proved that AF derivatives adhered also to human erythrocytes, which suggests that they may have cytotoxic activity towards eukaryotic cells [8]. On the other hand, some authors demonstrated that water-soluble fullerene derivatives linked with anticancer agents exhibited increased cytotoxicity towards cancer cells compared to free anticancer agents [24,25]. However, they evaluated the influence of obtained derivatives only towards cancer cells. Thus, it is possible that such amine derivatives of fullerene may also have unfavorable influence on normal cells.

It is known that cytocompatibility is a mandatory feature of potential medicinal products [40,41]. Thus, non-cytotoxic **60FGG** derivative seems to be promising candidate for further analysis.

## 3. Materials and Methods

**Figure 6:**
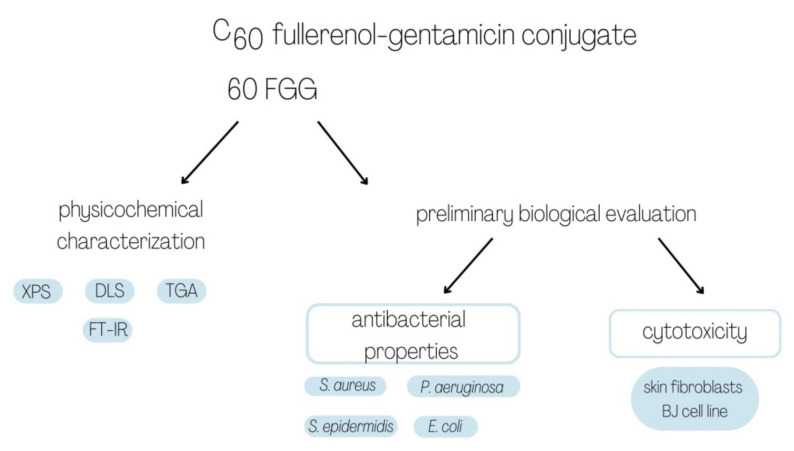
Schematic diagram presenting the experiments performed within this study.

The general plan of performed experiments was introduced in Figure 6.

### 3.1. Materials

Dimethylformamide (DMF), gentamicin sulfate salt, (3-glycidyloxypropyl)trimethoxysilane (GLYMO), methanol, penicillin-streptomycin solution, phosphate buffered saline (PBS), sodium dodecyl sulfate (SDS), sodium hydroxide, tetrabutylammonium hydroxide (TBAH), thiazolyl blue tetrazolium bromide (MTT), toluene, and trypsin-EDTA solution (0.25%) were obtained from Merck, Warsaw, Poland. Fetal bovine serum (FBS) was purchased from Pan-Biotech, Aidenbach, Germany. Eagle’s Minimum Essential Medium (EMEM), normal human skin fibroblasts (BJ cell line, CRL-2522^TM^), Staphylococcus aureus (ATCC 25923), Staphylococcus epidermidis (ATCC 12228), Pseudomonas aeruginosa (ATCC 27859), and Escherichia coli (ATCC 25922) were supplied by ATCC, Manassas, USA, while Mueller-Hinton agar (MHA) and Mueller-Hinton broth (MHB) by Oxoid, Hampshire, UK.

### 3.2. Synthesis and Characterization of C_60_ Fullerenol-Gentamicin Derivative

Gentamicin functionalized C_60_ fullerene (**60FGG**) was obtained in three-step synthesis. In the first step we obtained hydroxylated C_60_ using modified method reported by Li and Takeuchi [42]. Briefly, C_60_ (200 mg) was dissolved in 150 mL of toluene, then 10 mL of saturated NaOH solution was added, followed by addition of catalytic amount of TBAH. The solution was then stirred for an hour, until the organic layer became colorless and brown precipitate appeared. Toluene was decanted from the solid and 25 mL of deionized water was added. After the solution was stirred for additional 10 h, another 50 mL of water were added and the resultant mixture was filtered through a fluted filter. Obtained orange solution was concentrated on rotary evaporator to c.a. 20 mL and 125 mL of methanol was added to precipitate product. Afterwards, crude product was re-dissolved in deionized water and dialyzed (1 kDa cut off). Resultant solution was precipitated using methanol, centrifuged and dried in a vacuum oven. TGA measurements allowed to estimate the composition of synthesized fullerenol to be C_60_(OH)_24_.

**Figure 7:**
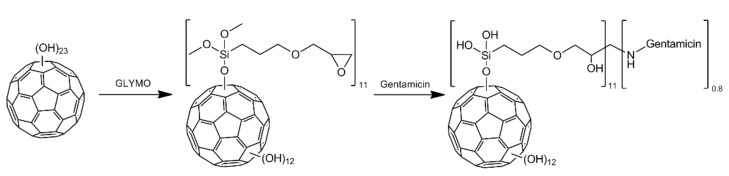
Scheme for synthesis of gentamicin functionalized C_60_ fullerene (**60FGG**) using fullerenol as a substrate.

The second step was concentrated on the addition of (3-glycidyloxypropyl)trimethoxysilane (GLYMO) to the hydroxyl groups previously introduced onto fullerene surface (Figure 7). 150 mg of synthesized fullerenol was dispersed in 12 mL of anhydrous toluene using ultrasound. Then 1.2 mL of GLYMO was added and resultant mixture was refluxed in argon atmosphere for 24 h. Then, methanol was added and mixture was centrifuged. The liquids were decanted from the solid, which was washed with methanol and then dried in vacuo.

The final step was performed using the modified method reported by Lizza et al. [43], where epoxide ring was opened using primary amino groups from gentamicin molecules. Crude GLYMO functionalized fullerene **60FG** (65 mg, 0.02 mmoL) was sonicated in 8 mL of DMF, followed by addition of gentamicin (94 mg, 0.2 mmol) dissolved in small amount of DMF. The reaction was carried out at 60 °C for 24 h. The water (18 mL, 1 mmoL) was added and the mixture was centrifuged to separate insoluble residue. Obtained brown liquid was decanted and dialyzed (1 kDa cut off). Then, it was concentrated on rotary evaporator and methanol was added in order to form a precipitate of the final product, which composition was estimated to be C_60_(OH)_12_(GLYMO)_11_(Gentamicin)_0.8_.

Obtained products were characterized using X-ray photoelectron spectroscopy (XPS), Fourier-transform infrared spectroscopy (FT-IR), and thermogravimetry (TGA). XPS measurements were carried out using a VG ESCALAB 210 electron spectrometer (Thermo Fisher Sceintific, Bothell, WA, USA) equipped with an Al-Kα source (1486.6 eV). XPS data were calibrated using the binding energy of C1s = 284.6 eV as the internal standard. The infrared experiments were performed on the Shimadzu FTIR-8400S (Shimadzu, Kioto, Japan). The samples were prepared as KBr disks. Thermogravimetric measurements were performed using Q50 TGA (TA Instruments, New Castle, DE, USA). The analyzed sample was previously dried under vacuum at 60 °C and the measurement was carried out under flow of nitrogen with heating rate of 5 K/min. Size of synthesized fullerene derivatives their Zeta potential were measured in water using Zetasizer Nano (Malvern, UK).

### 3.3. Biological Properties of PEG Functionalized C_60_ Fullerenol-Gentamicin Conjugate

For biological assays, the following samples were used: C_60_ fullerenol (**60F**), gentamicin sulfate salt (**G**), and C_60_ fullerenol-gentamicin conjugate (**60FGG**). The samples were put into 1.5 mL eppendorf tubes and sterilized by ethylene oxide. During preparation of aqueous solutions of samples (in sterile deionized water), estimated structure of synthesized fullerene derivative (C_60_(OH)_12_(GLYMO)_11_(Gentamicin)_0.8_) was taken into account. It was assumed that **60FGG** contains 88 wt.% of modified C_60_ fullerenol and 12 wt.% of gentamicin. Thus, tested concentrations of **60F** and **G** in solutions of **60FGG** ranged 29.25–0.057 μg/mL and 4–0.007 μg/mL, respectively. In parallel, **60F** was tested at concentrations ranged 29.25–0.057 μg/mL, while **G** at concentrations ranged 4–0.007 μg/mL.

#### 3.3.1. Antibacterial Properties In Vitro

In order to evaluate antibacterial activity of tested samples, minimum inhibitory concentration (MIC) and minimum bactericidal concentration (MBC) were determined. The MIC was estimated using microdilution method according to the CLSI M7A7 standard [44]. Briefly, the two-fold dilutions of tested samples were prepared in MHB broth. Then, 100 μL of solutions were added to 96-well plate. 100 μL of MHB was used as a control solution. The bacteria inoculum at 0.5 McFarland standard density (1 × 10^8^ CFU/mL) was prepared in sterile 0.85% saline using nephelometer (BD PhoenixSpec^TM^ Nephelometer, Thermo Fisher Scientific, Waltham, WA, USA). Then, inoculum was 200-times diluted in order to obtain 5 × 10^5^ CFU/mL. 100 μL of diluted inoculum was added to 100 μL of tested sample solutions or MHB (control), which were placed in 96-well plate. The plates were incubated in air at 37 °C without agitation for 24 h. Then, the absorbance was read at 600 nm (Synergy H4 automatic plate reader, BioTek, Winooski, VT, USA). The experiment was performed in three separate measurements in triplicate. The MIC was defined as the lowest compound concentration that inhibits more than 90% of bacterial growth. Then, in order to determine MBC, 100 μL of each well without bacterial growth was seeded on Petri dish containing MHA. After 24 h incubation at 37 °C without agitation, the colonies were count. MBC was defined as the lowest compound concentration that decreased the number of colonies by ≥99.99% compared to the control growth. The experiment was performed in three separate replicates.

#### 3.3.2. Cytotoxic Properties In Vitro

Cytotoxicity of compounds was evaluated using normal skin fibroblasts (BJ cell lines) as described in details previously [45]. Briefly, BJ cells were seeded in 96-well plate at concentration of 2 × 10^4^ cell/well and incubated at 37 °C for 24 h. On the next day, the two-fold dilutions of tested samples were prepared in EMEM medium. Then, cell medium from above the cells was gently removed and 100 μL of prepared solutions were added. 100 μL of fresh EMEM medium was used as a control solution. After 24 h incubation at 37 °C, fibroblast viability was assessed using MTT assay. The experiment was performed in three separate measurements in octuplicate.

## 4. Conclusions

In this study, (3-glycidyloxypropyl)trismethoxysilane functionalized fullerene (**60FG**) was used as a vehicle for a standard aminoglycoside antibiotic–gentamicin (**G**). It is worth underlining that this is the first study which shows effective method to conjugate fullerenol with aminoglycoside antibiotic. We believe that such conjugate may allow to link biological properties of fullerenol (for instance antioxidant or free-radical scavenging) and antibacterial properties of gentamicin. It seems to be very important since the application of antibiotics (such as gentamicin) is associated with unfavorable generation of free radicals, which may lead to nephrotoxicity. Our preliminary results demonstrated that applied three-step fabrication procedure allowed to obtain gentamicin functionalized C_60_ fullerene (**60FGG**), as proven by XPS analysis, termogravimetric evaluation, and FT-IR measurements. Obtained **60FGG** derivative exhibited beneficial antibacterial activity against Gram-positive and Gram-negative bacterial strains, indicating that such conjugate enabled the maintaining of inhibition activity of gentamicin. Moreover, cell culture experiments proved that **60FGG** derivative did not possess cytotoxic properties towards normal human fibroblasts (model eukaryotic cells). From biomedical point of view, these preliminary results are very promising and allow to consider further application of obtained **60FGG** derivative. For instance, obtained fullerenol-gentamicin conjugate could be potentially used as injection solution with antibacterial properties for the treatment of chronic wounds or osteochondral defects. It may be also applied as a component of bioactive biomaterials, such as wound dressings or implantable biomaterials, which are prone to bacterial infections. Presumably, addition of fullerenol-gentamicin conjugate to biomaterial, instead of gentamicin alone, allow to obtain construct possessing ability to release antibiotic in controllable manner and hopefully with antioxidant properties, thanks to presence of C_60_ fullerenol. Nevertheless, in order to verify these hypotheses, additional experiments are needed. Thus, we plan to expand our research involving (e.g., evaluation of antioxidant properties of 60FGG derivative and possibility to use it as a component of bioactive biomaterials).

## Figures and Tables

**Table 1:** MIC values for *S. aureus*, *S. epidermidis*, *P. aeruginosa*, and *E. coli* after 24 h incubation with aqueous solutions of gentamicin sulfate salt (**G**), C_60_ fullerenol (**60F**), and gentamicin functionalized C_60_ fullerene (**60FGG**).

Bacteria	Minimum Inhibitory Concentration (MIC) ^a^ μg/mL		
	G	60F	60FGG
*S. aureus* ATCC 25923	0.125	ND ^b^	0.125
*S. epidermidis* ATCC 12228	0.25	ND ^b^	0.25
*P. aeruginosa* ATCC 27859	0.25	ND ^b^	0.25
*E. coli* ATCC 25922	1	ND ^b^	1

^a^ Minimum Inhibitory Concentration (MIC)–the lowest compound concentration that inhibits more than 90% of bacterial growth. ^b^ ND (not determined). In tested concentrations, no antibacterial activity was observed.

**Table 2:** MBC values for *S. aureus*, *S. epidermidis*, *P. aeruginosa*, and *E. coli* after treatment with aqueous solutions of gentamicin sulfate salt (**G**), C_60_ fullerenol (**60F**), and gentamicin functionalized C_60_ fullerene (**60FGG**).

Bacteria	Minimum Bactericidal Concentration (MBC) ^a^ μg/mL		
	G	60F	60FGG
*S. aureus* ATCC 25923	0.25	NT ^b^	0.25
*S. epidermidis* ATCC 12228	0.5	NT ^b^	0.5
*P. aeruginosa* ATCC 27859	0.25	NT ^b^	0.25
*E. coli* ATCC 25922	1	NT ^b^	1

^a^ Minimum Bactericidal Concentration (MBC)–the lowest compound concentration that decreased the number of colonies by ≥99.99% compared to the control growth. ^b^ NT (not tested). Lack of antibacterial activity was determined already during MIC evaluation.

## Data Availability

Not applicable.

## Supplementary Materials

The following supporting information can be downloaded at: https://www.mdpi.com/article/10.3390/molecules27144366/s1, Figure S1: Survey XPS spectrum of gentamicine modified C_60_ fullerene (**60FGG**), Figure S2: Zeta potential measurements of C_60_ fullerenol (**60F**), Figure S3: Zeta potential measurements of gentamicin functionalized C_60_ fullerene (**60FGG**).

## Sample Availability

Samples of C_60_ fullerenol-gentamicin derivative are available from the authors.
