# Supplementary material for: Novel C60 Fullerenol-Gentamicin Conjugate–Physicochemical Characterization and Evaluation of Antibacterial and Cytotoxic Properties
Source: Molecules. 2022 Jul 7;27(14):4366. doi: 10.3390/molecules27144366 (PMC9317625; doi:10.3390/molecules27144366)
Supplement: Supplementary file 1 [file molecules-27-04366-s001.zip › molecules-1768350-supplementary.pdf]

Supplementary Material

Novel C_{60} Fullerenol-Gentamicin Conjugate–Physicochemical Characterization and Evaluation of Antibacterial and Cytotoxic Properties

Aleksandra Nurzynska [1], Piotr Piotrowski [2,*], Katarzyna Klimek [1,*], Julia Król [2], Andrzej Kaim, [2] and Grazyna Ginalska [1]

[1] Chair and Department of Biochemistry and Biotechnology, Medical University of Lublin, Chodzki 1 Street, 20-093 Lublin, Poland; aleksandra.nurzynska@umlub.pl (A.N.); g.ginalska@umlub.pl (G.G.)
[2] Department of Chemistry, University of Warsaw, Pasteura 1 Street, 02-093 Warsaw, Poland; j.krol10@student.uw.edu.pl (J.K.); akaim@chem.uw.edu.pl (A.K.)
* Correspondence: ppiotrowski@chem.uw.edu.pl (P.P.); katarzyna.klimek@umlub.pl (K.K.)

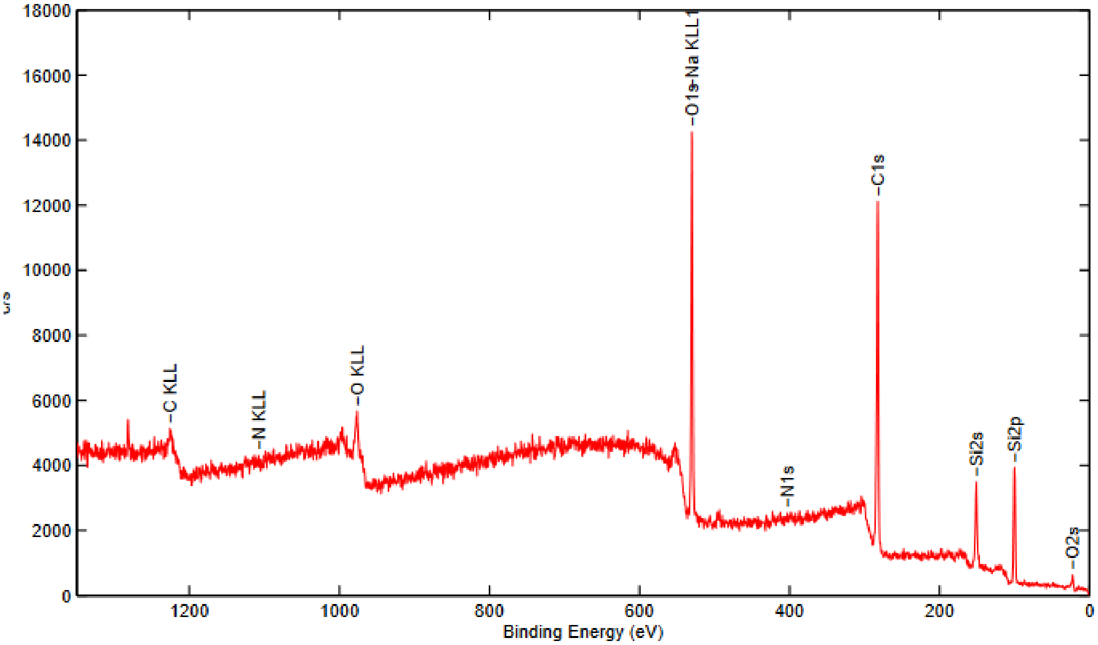

Figure S1. Survey XPS spectrum of gentamicine modified C_{60} fullerene (**60FGG**).

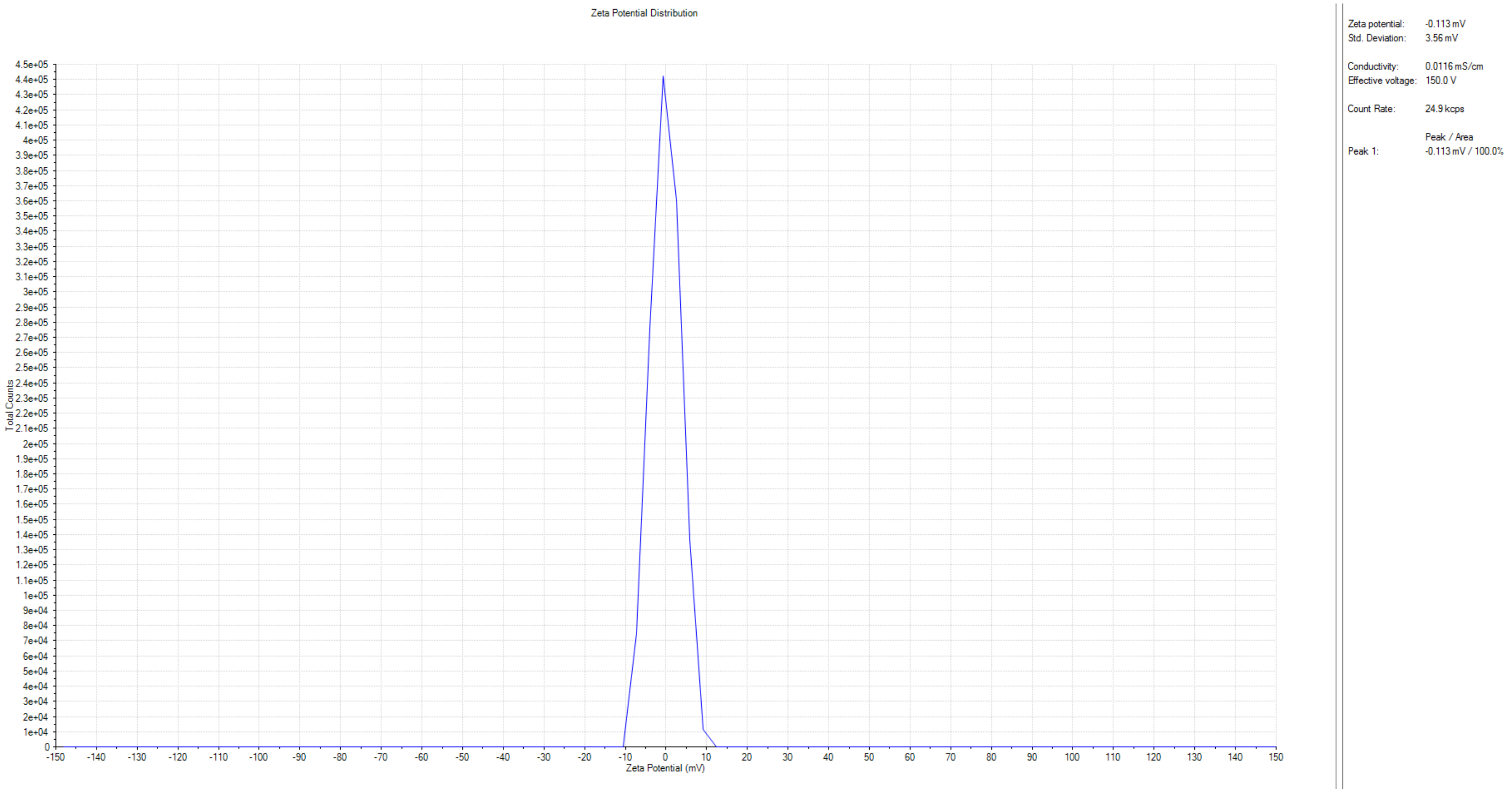

Figure S2. Zeta potential measurements of C_{60} fullerenol (**60F**).

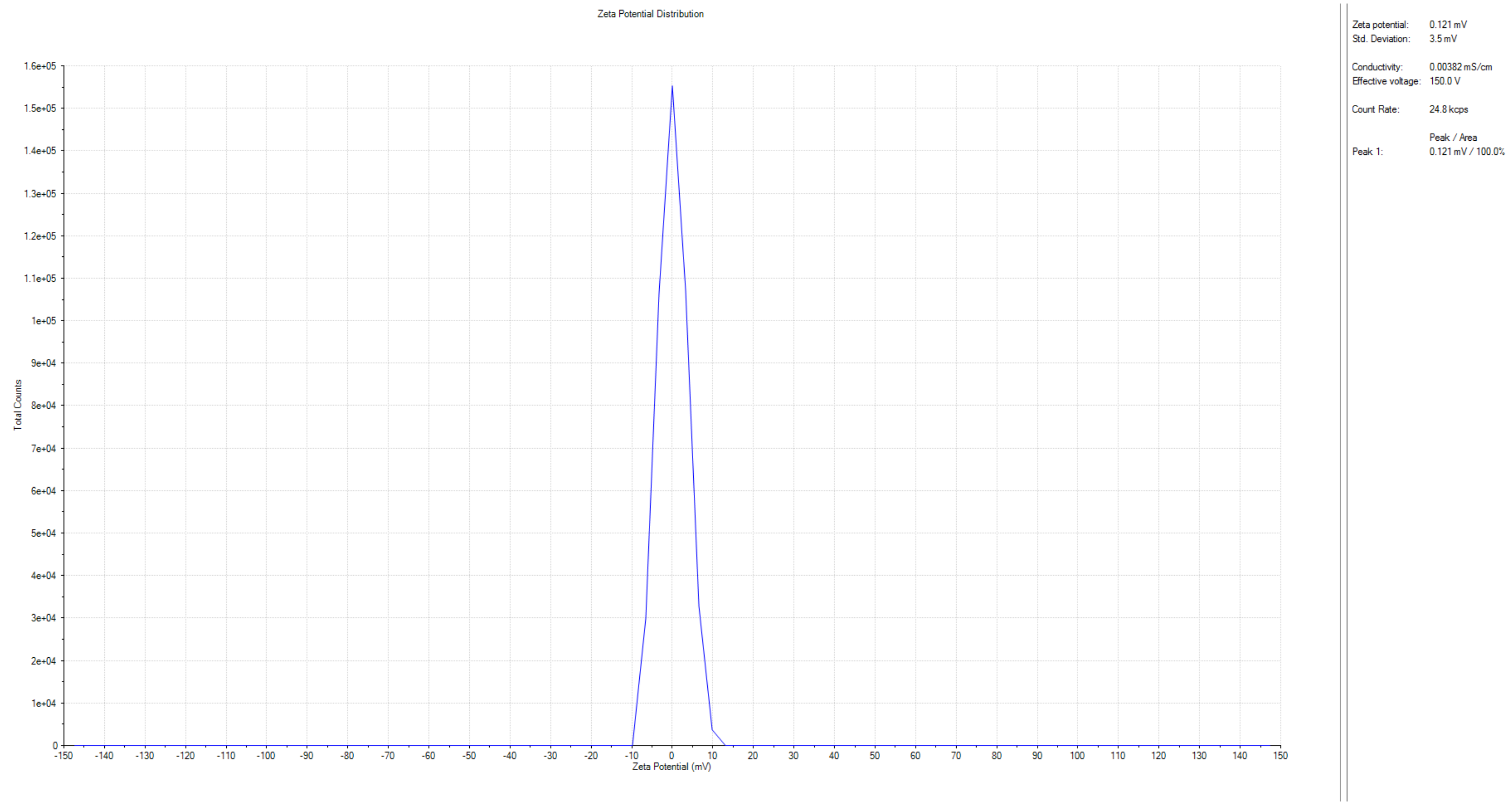

Figure S3. Zeta potential measurements of gentamicin functionalized C_{60} fullerene (**60FGG**).
